# Aesthetic Processing Differences Between Representational and Defamiliarized Photographic Images During Initial and Repeated Presentations: Evidence from ERPs

**DOI:** 10.3390/brainsci16101018

**Published:** 2026-09-24

**Authors:** Jinru Liu, Xiaoxia Wang, Jie Zhang, Jianqing Tuo

**Affiliations:** 1 School of Humanities and Social Science, Xi’an Jiaotong University, Xi’an 710049, China; 565337022@stu.xjtu.edu.cn; 2 The School of Film and Television Art, Hebei Institute of Communications, Shijiazhuang 051430, China; 3 School of Foreign Languages, Hebei University of Economics and Business, Shijiazhuang 050062, China; wangx2@hueb.edu.cn; 4 Department of Psychology, Hebei Normal University, Shijiazhuang 050024, China

**Keywords:** initial and repeated, defamiliarization, photography, aesthetic processing, FN400, LPP

## Abstract

**Highlights:**

**What are the main findings?**

- Representational photographic images were judged as “beautiful” more often than defamiliarized photographic images, but the second presentation did not significantly change explicit aesthetic judgments.
- ERP results showed that repetition effects varied by image type and processing stage: for representational photographic images, the second presentation elicited a more positive FN400 amplitude and significant reductions in the middle and late LPP time windows; for defamiliarized photographic images, the difference between the second and first presentations did not reach statistical significance.

**What are the implications of the main findings?**

- Explicit aesthetic judgments did not change significantly, whereas ERP responses showed repetition-related modulation, suggesting that ERP measures may be more sensitive than behavioral judgments to repetition-related changes in this task.
- Repeated presentation does not simply enhance or reduce neural processing; its effects may depend on image representationality and processing stage. This may offer preliminary insights into the debate between the mere exposure effect and aesthetic fatigue.

**Abstract:**

Objectives: Repeated presentation may enhance processing fluency, but as novelty wanes, it may also reduce viewers’ cognitive engagement. Given that representational photographic images (RPIs) and defamiliarized photographic images (DPIs) differ in semantic clarity, it remains unclear whether repeated viewing differentially affects viewers’ explicit aesthetic judgments and neural processing during aesthetic judgments of these images. Methods: This study adopted event-related potentials (ERPs), and examined 40 participants’ aesthetic judgments of RPI and DPI during initial and repeated presentation. The behavioral measure was the proportion of images judged as “beautiful,” and ERP analyses focused on FN400 and early (500–1200 ms), mid (1200–3000 ms), and late (3000–5000 ms) LPP components. Results: Behavioral results showed that RPIs were judged as “beautiful” more often than DPIs, and presentation order did not significantly alter explicit aesthetic judgments. ERP results showed that repetition effects differed by image type: for RPIs, FN400 amplitudes were more positive and middle and late LPP amplitudes were reduced during the second presentation, whereas the repetition effect was not statistically significant for DPIs. Conclusions: Repeated presentation did not significantly alter explicit aesthetic judgments, but ERP repetition effects were observed mainly for RPIs and varied across processing stages. These preliminary findings suggest that the effects of repeated presentation on aesthetic judgments may be stage-dependent and stimulus-dependent. Future research should further disentangle presentation order from block order and more strictly match low-level visual features to test these effects.

## 1. Introduction

### 1.1. Defamiliarization Theory and Repeated Presentation

The theory of defamiliarization (ostranenie) was first proposed by Russian Formalist aesthetician Viktor Shklovsky. He argued the purpose of art is not to represent things in a habitual manner, but to break the automatism of everyday perception through the technique of “defamiliarization” (ostranenie), thereby restoring to people the direct experience of things as they are. In other words, artistic forms enhance aesthetic experience by complicating and prolonging the mode of expression, which increases the difficulty and duration of perception [1]. This theory has exerted a profound influence on photographic practice. Whether through Francis Bruguière’s (1879–1945) light experiments during the Modernist period or Alvin Langdon Coburn’s (1882–1966) vortographic experiments, all these artists sought to break through photography’s conventional representation of reality through formal experimentation, re-presenting the world in a defamiliarized visual manner [2,3]. However, while alienated artistic forms expand visual experience, they simultaneously reduce the cognitive fluency in aesthetic appreciation, making it difficult for viewers to grasp the meaning and comprehend the formal structure at first glance. As a result, repeated presentation and sustained engagement are often required to fully access the aesthetic structure of the work [4].

While repeated presentation may help viewers overcome initial comprehension barriers and deepen their familiarity with the form and meaning of the work [5,6], it may simultaneously diminish aesthetic interest as the novelty of the stimulus wanes [7].

### 1.2. The Dual Effects of Repeated Processing

Existing research on how repeated presentation affects aesthetic experience has primarily followed two lines of inquiry. One strand of research suggests that aesthetic ratings decline linearly overall with increasing repetition [8,9,10,11,12]. A second strand of research, by contrast, emphasizes that repeated presentation of a stimulus enhances individuals’ liking and preference for it [13,14,15]. These two sets of findings are not necessarily contradictory; rather, they may stem from differences in task demands and measurement contexts. Mere exposure effect studies typically employ a two-stage paradigm involving repeated presentation followed by subsequent evaluation, and the exposure phase does not require participants to make immediate aesthetic judgments [16]. In contrast, aesthetic fatigue studies are more concerned with changes in individuals’ evaluative responses over the course of continuous aesthetic judgments [9]. Therefore, in the context of successive aesthetic judgments, how aesthetic processing changes when the same stimulus is re-presented, and whether this change is modulated by stimulus type, remain to be further examined.

### 1.3. Limitations of Behavioral Measures and the Introduction of ERP Methodology

From a methodological standpoint, existing studies have largely relied on behavioral measures such as preference ratings and reaction times to investigate this issue. However, such measures are insufficient for fully revealing the temporal course of aesthetic processing and its underlying mechanisms, particularly under short-term repeated presentation conditions when individuals’ explicit aesthetic judgments may not yet show significant changes, whereas processes such as stimulus identification, meaning extraction, attentional resource allocation, and sustained evaluation may have already undergone adjustments. In other words, repetition effects may be first manifested as neural-level processing changes rather than in behavioral ratings.

Event-related potential (ERP) techniques, with their high temporal resolution, can precisely capture the temporal dynamics of aesthetic processing at different stages, providing more fine-grained neural evidence for understanding repeated aesthetic processing [17,18]. Drawing on the dual-process model of recognition memory [19], repeated presentation typically modulates both familiarity and recollection processes, which are often reflected in ERP studies as differential responses in the FN400 and Late Positive Potential (LPP) components. The FN400 typically reflects conceptual priming or facilitated conceptual processing resulting from repetition [20], and thus serves as a key index of repeated processing and familiarization. Following repeated stimulus presentation, once a stable mental representation has been established, subsequent encounters may elicit a reduced FN400 amplitude. The late positive potential (LPP) usually reflects sustained attentional engagement, emotional intensity, motivational significance, and higher-order cognitive evaluation [21,22]. In aesthetic judgment tasks, the LPP can be considered a key neural indicator of an individual’s sustained allocation of aesthetic resources. If repeated presentation leads to a subsequent attenuation of the LPP, it may suggest a reduced need for further evaluation or a decreased engagement in aesthetic processing.

### 1.4. Stimulus Type Differences

Different types of aesthetic stimuli may exhibit distinct processing trajectories during repeated presentation. Representational photographic images typically contain relatively stable object cues and semantic information, and are therefore more likely to form clear memory representations. Accordingly, during the second presentation, these stimuli are more likely to show neural changes associated with increased familiarity and enhanced processing fluency [23]. In contrast, defamiliarized photographic images typically lack clear object cues and stable semantic information, making their processing more dependent on formal features, perceptual organization, and viewers’ subjective construction [24]. Therefore, during the second presentation, defamiliarized photographic images may not develop a stable sense of familiarity as rapidly as representational photographic images. Because their meanings are often not fixed after a single viewing, these images may retain greater interpretive space during successive aesthetic judgments, thereby maintaining relatively stable processing engagement. However, such stimulus-type-related processing differences may not be directly reflected in explicit aesthetic judgments, because behavioral measures have limited sensitivity to differences across processing stages. On this basis, the present study used event-related potentials (ERPs), with aesthetic judgments as the behavioral measure and FN400 and early, middle, and late LPP as neural measures, to examine aesthetic processing of representational and defamiliarized photographic images during initial and repeated presentations, and to test whether the effect of repeated presentation on aesthetic processing was jointly modulated by stimulus type and processing stage.

### 1.5. Hypotheses

Based on the above discussion, this study proposes the following hypotheses:

**H1.**  *The effect of the second presentation on aesthetic processing will be manifested primarily in ERP components rather than in aesthetic judgments, and this ERP-level repetition effect will vary as a function of stimulus type.*

**H2.**  *Within the FN400 time window, the modulatory effect of the second presentation on ERP amplitude will differ between representational and defamiliarized photographic images, and this effect is expected to emerge primarily in the representational photographic image condition.*

**H3.**  *Within the middle and late LPP time windows, an interaction occurs between the second presentation and stimulus type. Specifically, whereas representational photographic images are expected to show a significant reduction in LPP amplitude during the second presentation, defamiliarized photographic images are not expected to show a similar reduction.*

## 2. Materials and Methods

### 2.1. Participants

The sample size was determined through an a priori using G*Power software (version 3.1.9.7) [25]. Referring to the effect size (*η*^2^*p* = 0.05) reported by Song et al. (2024) for between-group or between-condition differences in ERP studies, the G*Power analysis indicated that, with a significance level (α) of 0.05 and a statistical power (1 − β) of 0.90, a minimum of 36 participants were required to detect the anticipated effect [26].

A total of 44 participants were initially recruited for the ERP experiment. Data from four participants were excluded due to excessive muscle artifacts and electrode drift. The final sample thus comprised 40 participants (19 males and 21 females), with a mean age of 20.55 ± 1.11 years (mean ± SD). All participants had normal or corrected-to-normal vision, reported no history of neurological disease or cognitive impairment, and had not participated in any similar experiments. This study was approved by the Ethics Committee of Hebei Normal University. All participants were right-handed, had no history of psychiatric or neurological disorders, and had normal or corrected-to-normal vision. Prior to the experiment, all participants were fully informed of the study protocols and provided written informed consent.

### 2.2. Stimuli

The visual stimuli were digital photographs of architectural scenes, divided into two categories: representational photographic images (RPIs) and defamiliarized photographic images (DPIs). RPIs were single-exposure images without the superimposition or salient ghosting typically associated with multiple exposure. The RPIs were not the original single-exposure source images used to generate the DPIs but matched single-exposure control images. The RPIs and DPIs were matched on viewing angle, specifically an eye-level composition. DPIs were created with a Canon EOS 5D Mark III (Canon, Tokyo, Japan)using its built-in multiple-exposure function in overlay mode; each DPI comprised nine exposures.

For objective measurement, following the approach of Vissers et al. (2020), the Pyramid Histogram of Oriented Gradients (PHOG) was employed to assess image self-similarity [27]. PHOG reflects the structural consistency between local and global features by extracting the distribution of gradient orientations across multiple spatial scales [28]. The term “self-similarity” in this study does not refer to visual similarity in the general sense, but rather to the stability of edge-orientation organization across multiple spatial levels within an image [29]. Higher PHOG consistency typically indicates more stable contour organization and hierarchical structure, which may support object recognition and correlate with higher representationality. Conversely, lower PHOG consistency may suggest weaker structural organization and more ambiguous object contours, thereby increasing recognition uncertainty. Given that ambiguity and uncertainty are considered among the key mechanisms for eliciting defamiliarized aesthetic experiences [30,31], PHOG can serve as a low-level visual indicator for characterizing image structural stability and its potential defamiliarization effects.

Based on PHOG values, this study ultimately selected 40 photographs as stimulus materials (see Figure 1 for examples). Paired-samples t-tests revealed that PHOG values for DPI (M = 284.28, SD = 245.94) were significantly lower than those for RPI (M = 4632.33, SD = 3734.91). In this study, given that the raw PHOG values showed substantial dispersion and may have been skewed, the PHOG values were log-transformed (natural log), and the statistical analyses were rerun using ln(PHOG). A repeated-measures ANOVA showed that the ln(PHOG) values for DPIs (M = 4.85, SD = 1.66) were significantly lower than those for RPIs (M = 8.18, SD = 0.55), *F*_(1,19)_ = 70.64, *p* < 0.001, *η*^2^*p* = 0.788, indicating that the two types of stimuli were preliminarily differentiated in terms of image-structure stability and the clarity of object cues.

To confirm that the stimulus materials differed perceptually in terms of familiarization and defamiliarization, an independent sample of 26 participants who did not take part in the formal aesthetic processing experiment was recruited to rate all 40 images on the degree of familiarization. Ratings were made on a 7-point Likert scale, with 1 indicating “least defamiliarized” and 7 indicating “most defamiliarized.” Descriptive results showed that multiple-exposure photographs received significantly higher defamiliarization ratings (M = 6.15, SD = 0.26) than single-exposure photographs (M = 2.30, SD = 0.54), *F*_(1,25)_ = 29.04, *p* < 0.001, *η*^2^*p* = 0.540, indicating good discriminant validity of the stimulus materials along the defamiliarization dimension.

This study further compared the basic physical properties of all experimental images, including contrast, mean luminance, and spatial frequency. Results showed no significant difference in contrast between representational photographic images (0.24 ± 0.06) and defamiliarized ones (0.23 ± 0.08) *F*_(1,38)_ = 0.22, *p* = 0.645. Similarly, no significant difference was found in mean luminance in single-exposure photographs at 0.15 ± 0.04 cd/m^2^ and multiple-exposure photographs at 0.14 ± 0.06 cd/m^2^, *F*_(1,38)_ = 0.08, *p* = 0.779. However, the two types of images differed significantly in spatial frequency. RPIs (0.24 ± 0.02 cycles/pixel) showed significantly higher spatial frequency than DPIs (0.20 ± 0.02 cycles/pixel), *F*_(1,38)_ = 21.36, *p* < 0.001, *η*^2^*p* = 0.360. This likely stems from the inherent characteristics of the multiple-exposure technique itself, as this technique tends to produce smoother pixel intensity transitions across spatial regions of the image, thereby attenuating high-frequency components.

### 2.3. Procedure

The experiment consisted of two blocks, each containing 40 trials, with 20 RPIs and 20 DPIs per block. Each image was presented twice: first in Block 1, then again in Block 2. Thus, the experiment proper comprised a total of 80 trials. Trial order within each block was randomized. All stimuli were grayscale images, sized at 800 × 800 pixels at 96 dpi resolution, and presented at the center of a black background.

On each trial, a fixation cross was first presented at the center of the screen for 1500 ms, followed by the target image for 5000 ms. After the image disappeared, a response phase began, during which participants were required to make an aesthetic judgment within 2000 ms based on their own perception of the image. If participants judged the photograph as “beautiful”, they pressed the “F” key; if they judged it as “not beautiful”, they pressed the “J” key. If a response was made within the allotted time, the program proceeded immediately to the next phase; if no response was made, the program automatically proceeded to the next phase after the 2000-ms response window elapsed. A blank screen interval of 1400–1600 ms followed. A 2-m break was provided between the two blocks. The experimental procedure is illustrated in Figure 2.

### 2.4. EEG Recording and Analysis

Electroencephalographic (EEG) signals were recorded using a 64-channel Ag/AgCl electrode cap fitted with a NeuroScan SynAmps system (Compumedics Neuroscan, Charlotte, NC, USA), with all electrodes positioned according to the international 10–20 system. EEG signals were digitized at a sampling rate of 500 Hz and were bandpass filtered online at 0.05–100 Hz. Four external electrodes were placed at the outer canthi of both eyes and above and below the left eye to record horizontal and vertical electrooculograms (EOG). During recording, the left mastoid served as the online reference; offline analyses were re-referenced to the average of the left and right mastoids. All electrode impedances were maintained below 5 kΩ.

EEG data were processed using EEGLAB (version v2025.0.0) [32] and ERPLAB (version v12.0.0) [33] toolboxes. First, EEG signals were digitally bandpass filtered at 0.05–40 Hz, followed by independent component analysis (ICA) to identify and remove components associated with eye movements and blinks. EEG was then segmented into 5200-ms epochs, spanning from 200 ms prior to stimulus onset to 5000 ms post-onset, with the –200 to 0 ms interval serving as the baseline for ERP component measurement. Artifact rejection was performed prior to averaging, with trials containing amplitudes exceeding ±100 µV excluded. After artifact rejection, the remaining numbers of trials were as follows: first presentation—RPI: 19 ± 1 trials, DPI: 19 ± 2 trials; second presentation—RPI: 18 ± 2 trials, DPI: 18 ± 2 trials.

### 2.5. Statistical Analysis

The behavioral measure was the proportion of trials in which participants judged an image as “beautiful.” To further test the robustness of the behavioral results, a generalized linear mixed-effects model (GLMM) with a binomial distribution and logit link was used to analyze the trial-level binary aesthetic judgments (“beautiful” vs. “not beautiful”). This model preserved trial-level binary responses while controlling for variability at the participant and image-item levels. A total of 3167 valid trials were included. Fixed effects were stimulus type (RPI vs. DPI), presentation order, and their interaction, with participants and image items included as random intercepts.

ERP analyses focused on the FN400 component and the early, middle, and late phases of the late positive potential (LPP). Descriptive statistics for all ERP measures are presented in Table 1. The selection of these ERP components and time windows was based on the typical latency ranges reported in previous studies. This selection was confirmed before analysis using grand-average waveforms and topographic maps collapsed across all experimental conditions. Specifically, based on the typical frontal distribution of the FN400 reported in previous studies, five frontal electrodes (AF3, AF4, F1, FZ, F2) were selected for the analysis of the FN400 component [34,35,36], while six centroparietal electrodes (C1, CZ, C2, CP1, CPZ, CP2) were selected for the LPP analysis [37,38,39]. The FN400 was quantified as the mean amplitude within the 300–500 ms time window. The LPP mean amplitude was further analyzed across three consecutive time windows: 500–1200 ms (early LPP), 1200–3000 ms (middle LPP), and 3000–5000 ms (late LPP), in accordance with prior studies [40,41].

Grand-average ERP waveforms for the 1-s and 5-s post-stimulus periods are depicted in Figure 3 and Figure 4, respectively.

Statistical analyses were conducted using IBM SPSS Statistics (version 26.0). A series of 2 × 2 repeated-measures analyses of variance (ANOVAs) were performed on the proportion of “beautiful” responses and the mean amplitudes of the FN400, early LPP, middle LPP, and late LPP components. Presentation order (two levels: first presentation vs. second presentation) and stimulus type (two levels: Representational Photographic Images vs. Defamiliarized Photographic Images) were both within-subjects factors. The significance level was set at α = 0.05.

For significant main effects and interactions, we conducted post hoc pairwise comparisons using Bonferroni correction. All post hoc *p*-values reported in the text are Bonferroni corrected.

## 3. Results

### 3.1. Behavioral Results

#### Aesthetic Judgment

Results revealed a significant main effect of stimulus type, *F*_(1,39)_ = 13.07, *p* < 0.001, *η*^2^*p* = 0.251, indicating that representational photographic images were judged as “beautiful” at a significantly higher proportion than defamiliarized photographic images (descriptive results are presented in Table 1). The main effect of presentation order was not significant, *F*_(1,39)_ = 0.003, *p* = 0.955. The stimulus type × presentation order interaction was also not significant, *F*_(1,39)_ = 2.63, *p* = 0.113.

To further account for the binary nature of the “beautiful” judgments and random variability across participants and image items, we used a generalized linear mixed-effects model. RPIs were judged as “beautiful” with significantly higher odds than DPIs; after controlling for random variability across participants and image items, the odds of a “beautiful” response for RPIs were approximately 3.81 times those for DPIs. The main effect of presentation order was not significant, and the interaction between stimulus type and presentation order was also not significant. GLMM results are presented in Table 2.

### 3.2. ERP Results

#### 3.2.1. FN400

The main effect of stimulus type was not significant, *F*_(1,39)_ = 1.06, *p* = 0.309. The main effect of presentation order was significant, *F*_(1,39)_ = 17.86, *p* < 0.001, *η*^2^*p* = 0.314, indicating that the amplitude elicited by the second presentation was overall more positive than that elicited by the first presentation. The Condition × Presentation Order interaction was significant, *F*_(1,39)_ = 4.69, *p* = 0.036, *η*^2^*p* = 0.107. Bonferroni-corrected simple effects analysis revealed that for the representational photographic images, the FN400 amplitude elicited by the second presentation was significantly more positive than that elicited by the first presentation, *p* < 0.001; for the defamiliarized photographic images, the difference between the first and second presentations did not reach statistical significance, *p* = 0.076. Further comparisons between the two image types within the same presentation order showed that the difference between representational and defamiliarized photographic images was not significant during the first presentation, *p* = 0.087; the difference between the two image types was also not significant during the second presentation, *p* = 0.875.

#### 3.2.2. Early LPP Component

The main effect of stimulus type was significant, *F*_(1,39)_ = 23.12, *p* < 0.001, *η*^2^*p* = 0.372, indicating that defamiliarized photographic images elicited more positive-going amplitudes than representational photographic images. The main effect of presentation order was not significant, *F*_(1,39)_ = 0.48, *p* = 0.491; the Condition × Presentation Order interaction was also not significant, *F*_(1,39)_ = 0.01, *p* = 0.919.

#### 3.2.3. Middle LPP Component

The main effect of stimulus type was not significant, *F*_(1,39)_ = 3.21, *p* = 0.081. The main effect of presentation order was significant, *F*_(1,39)_ = 12.60, *p* < 0.001, *η*^2^*p* = 0.244, indicating that amplitudes elicited by the first presentation were more positive than those elicited by the second presentation. The Condition × Presentation Order interaction was significant, *F*_(1,39)_ = 4.55, *p* = 0.039, *η*^2^*p* = 0.104. Bonferroni-corrected post-hoc tests revealed that under the representational photographic condition, amplitudes elicited by the first presentation were significantly more positive than those elicited by the second presentation (*p* < 0.001), whereas no such difference was observed for the defamiliarized photographic images (*p* = 0.656). Furthermore, during the second presentation, defamiliarized photographic images elicited significantly more positive amplitudes than representational photographic images (*p* = 0.009), but no such difference was found during the first presentation (*p* = 0.765).

#### 3.2.4. Late LPP Component

The main effect of stimulus type was not significant, *F*_(1,39)_ = 0.02, *p* = 0.882. The main effect of presentation order was significant, *F*_(1,39)_ = 18.90, *p* < 0.001, *η*^2^*p* = 0.326, indicating that amplitudes elicited by the first presentation were more positive than those elicited by the second presentation. The Condition × Presentation Order interaction was significant, *F*_(1,39)_ = 10.78, *p* = 0.002, *η*^2^*p* = 0.217. Bonferroni-corrected post-hoc tests revealed that for representational photographic images, amplitudes elicited by the first presentation were significantly more positive than those elicited by the second presentation (*p* < 0.001), whereas no such difference was observed for defamiliarized photographic images (*p* = 0.133). No significant amplitude differences between representational and defamiliarized photographic images were observed during either the first presentation (*p* = 0.240) or the second presentation (*p* = 0.204).

## 4. Discussion

This study used event-related potentials (ERPs) to investigate behavioral and neural differences in aesthetic judgments of RPIs versus DPIs across two repeated presentations. The behavioral results showed that RPIs were judged as “beautiful” significantly more often than DPIs, and this difference remained consistent across the first and second presentations. The second presentation did not significantly alter participants’ explicit aesthetic judgments, and this pattern was observed for both image types. These findings suggest that, under the experimental conditions of the present study, differences in immediate explicit aesthetic judgments were more closely associated with how representational the images were.

From a theoretical perspective, this result does not provide evidence for the explanation that repeated presentation leads to enhanced aesthetic evaluation. If the increase in familiarity induced by the second presentation were sufficient to alter aesthetic judgments, then the proportion of “beautiful” responses should have increased for both types of images after the second presentation; or at least the proportion of “beautiful” judgments for DPIs should have shown a clear increase after the second presentation. However, the behavioral results are more consistent with the interpretation that repeated presentation did not enhance explicit aesthetic judgments. This finding is in line with the results reported by Mikuni et al. (2021), who found that repeated presentation did not lead to an improvement in aesthetic evaluation [10].

The ERP results further showed that the modulatory effect of the second presentation on the two image types emerged at different stages of processing. Within the FN400 time window, the second presentation elicited overall more positive amplitudes, and this change was primarily observed in the RPI condition. During the LPP stage, differences between image types became progressively clearer across successive time windows. In the early LPP window, defamiliarized photographic images elicited overall more positive amplitudes, indicating that neural differences related to image type had already emerged at this stage; however, this difference was not clearly modulated by the second presentation. It should be noted that RPIs and DPIs differed significantly in spatial frequency. Given the potential sensitivity of the early LPP to low-level visual properties, the larger early LPP elicited by DPIs than by RPIs may also partly reflect these spatial-frequency differences. In the middle and late LPP windows, the amplitudes elicited by RPIs were significantly reduced during the second presentation relative to the first presentation, whereas DPIs did not show a comparable decrease. Notably, in the middle LPP window, DPIs elicited significantly larger amplitudes than RPIs during the second presentation. As shown by the waveforms and scalp topographies (Figure 4), the second presentation did not generally enhance ERP responses; rather, its effects were jointly modulated by image type and processing stage.

These findings provide more fine-grained evidence for understanding the neural mechanisms underlying visual aesthetics.

First, no significant repetition effect was observed in behavioral judgments, whereas the ERP results revealed stage-specific changes. This suggests that the influence of a second presentation on the aesthetic processing of the two types of images may not be immediately reflected in explicit aesthetic judgments, but may instead first emerge as changes in ERP responses. This result is consistent with Hypothesis H1 of the present study.

Second, the FN400 results indicate that the modulatory effect of the second presentation on neural activity within the FN400 time window was observed primarily in the RPIs condition, and this modulation was related to image type and presentation order. This finding was consistent with the H2 prediction. Importantly, the FN400 is not functionally identical to the semantic N400 [36]. Previous studies suggest that, in repetition paradigms, a more positive FN400 amplitude may reflect increased processing fluency, particularly enhanced conceptual fluency [42]. Thus, the more positive FN400 observed for RPIs during the second presentation may indicate a repetition priming effect. In contrast, DPIs did not show a significant FN400 repetition effect. There are at least two possible explanations for this non-significant effect. First, DPIs may involve relatively more ambiguous object representations and more open-ended meanings, making the second presentation insufficient to elicit detectable priming. Second, their lower recognizability may have weakened initial encoding, thereby reducing subsequent repetition priming.

Third, the LPP results revealed that the modulatory effect of second presentation on the two types of photographic images was primarily manifested in the middle and late time windows, and was not explicitly reflected in immediate aesthetic evaluation behavior. This LPP pattern suggested that the influence of second presentation on middle- and late-stage ERP activity was moderated by image type. Prior studies have demonstrated that the LPP is generally sensitive to sustained attention, motivational significance, and evaluative processing [21,43]. Thus, the significant changes in middle- and late-LPP components observed for RPIs upon second presentation may be modulated by sustained attention or evaluative processing. In contrast, defamiliarized photographic images did not show a similar decrease in LPP components. This result is consistent with the prediction of H3, namely that DPIs, compared with RPIs, maintained relatively stable middle- and late-LPP responses following the second presentation. This pattern is also compatible with Shklovsky’s view that defamiliarization may prolong the perceptual process, although the correspondence between the two remains to be further examined in future research.

Whether the effect of repeated exposure on aesthetic experience manifests as enhanced preference or aesthetic attenuation has long been a debate in the literature. The mere exposure effect emphasizes that familiarity and processing fluency may lead to increased preference [44,45]. In contrast, the aesthetic satiation perspective emphasizes that repeated presentation may lead to decreased novelty and reduced processing engagement [10,46,47]. This study suggests that the effect of repeated presentation is not unidirectional, but is jointly modulated by stimulus type and processing stage. The immediate aesthetic features of representational photographic images may derive more from clear cues and semantic recognizability, while defamiliarized photographs lie not in immediate aesthetic ratings, but rather in their ability to maintain relatively high neural processing engagement even after repeated presentation. This indicated that neural indices may be more sensitive than explicit aesthetic judgments. This is consistent with aesthetic processing models, which posit that aesthetic experience is not a single evaluative outcome, but is composed of multiple interactive processing components, including perceptual fluency, meaning construction, affective evaluation, and cognitive mastery [5,48].

## 5. Limitations

This study has several limitations. First, each image was presented only twice in the present study. This design makes it difficult to fully disentangle repetition effects from the potential influences of task duration, fatigue, habituation, or general task adaptation. Future studies could employ counterbalanced designs or mixed repetition sequences to further clarify the independent contributions of repetition-related processing and block-order effects.

Second, the present study employed a binary aesthetic judgment task; therefore, the behavioral measure reflected the proportion of images judged as “beautiful,” rather than continuous changes in the intensity of aesthetic experience. This may have reduced the sensitivity of the behavioral measure to repetition effects. In addition, differences in low-level visual features, such as spatial frequency, may have exerted some influence on late ERP components. Future studies should implement more rigorous control of low-level visual features and include steady-state visual control conditions to better dissociate low-level sensory processing from higher-level aesthetic evaluation. Moreover, 7-point or 9-point rating scales should be used to assess multiple subjective dimensions, including beauty, familiarity, novelty, complexity, comprehension difficulty, and emotional arousal, thereby allowing a more comprehensive examination of how repeated presentation affects distinct aspects of subjective experience.

Finally, the number of valid trials for the late LPP was relatively low, and given that slow-wave measures over multi-second windows are noisy, these trial counts may yield unstable single-condition estimates. Future studies could further improve the stability of ERP findings and validate these preliminary findings by more closely matching the spatial frequency spectra of the stimuli, incorporating steady-state visual control conditions, increasing the number of valid trials, or reporting split-half reliability.

## 6. Conclusions

RPIs and DPIs showed distinct ERP changes related to visual aesthetic processing across the first and second presentation blocks. The behavioral results showed that, in the first-presentation condition, RPIs were more likely to be judged as “beautiful” than DPIs. The second presentation did not significantly alter aesthetic judgments. The ERP results showed that, for RPIs, the FN400 became more positive and the middle and late LPP amplitudes decreased during the second presentation; DPIs did not show a similarly significant decrease. This pattern of amplitude changes suggests that the effects of the second presentation on processing related to aesthetic judgment may vary as a function of stimulus type and processing stage. However, given that presentation order was confounded with block order, that the number of valid trials for the late LPP was limited, and that low-level visual features were not fully matched, these findings should be interpreted as preliminary neural evidence. 

## Figures and Tables

**Figure 1:**
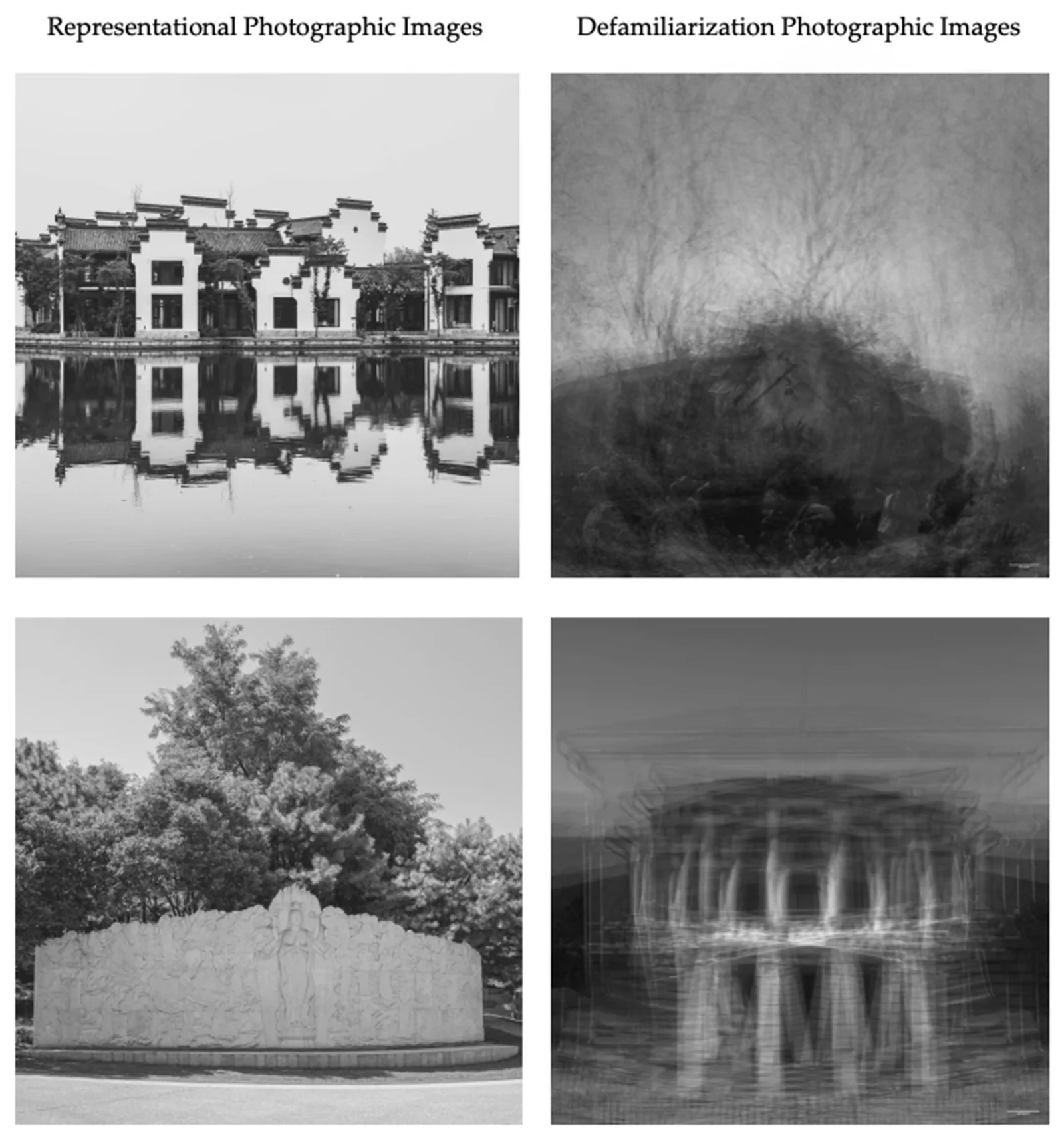
Representative examples of representational photographic images and defamiliarized photographic images.

**Figure 2:**
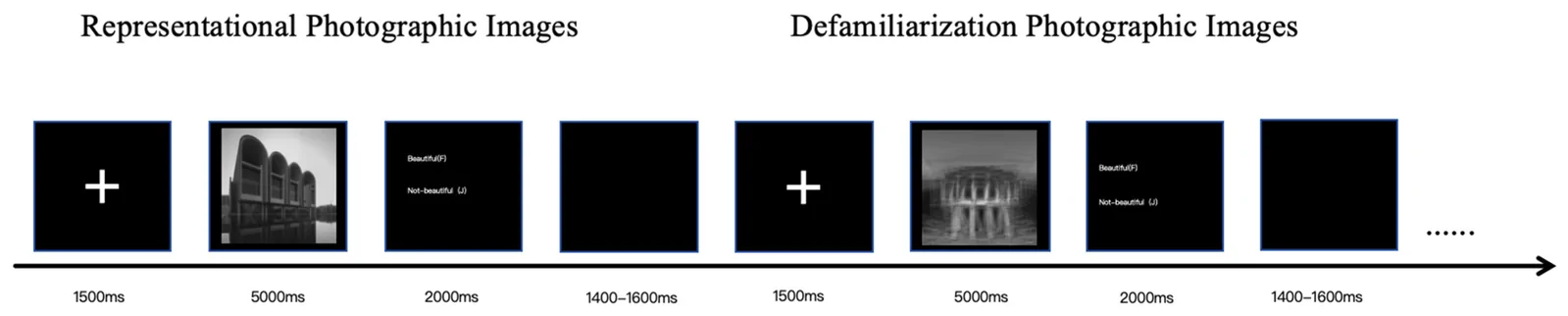
Schematic representation of the aesthetic processing task.

**Figure 3:**
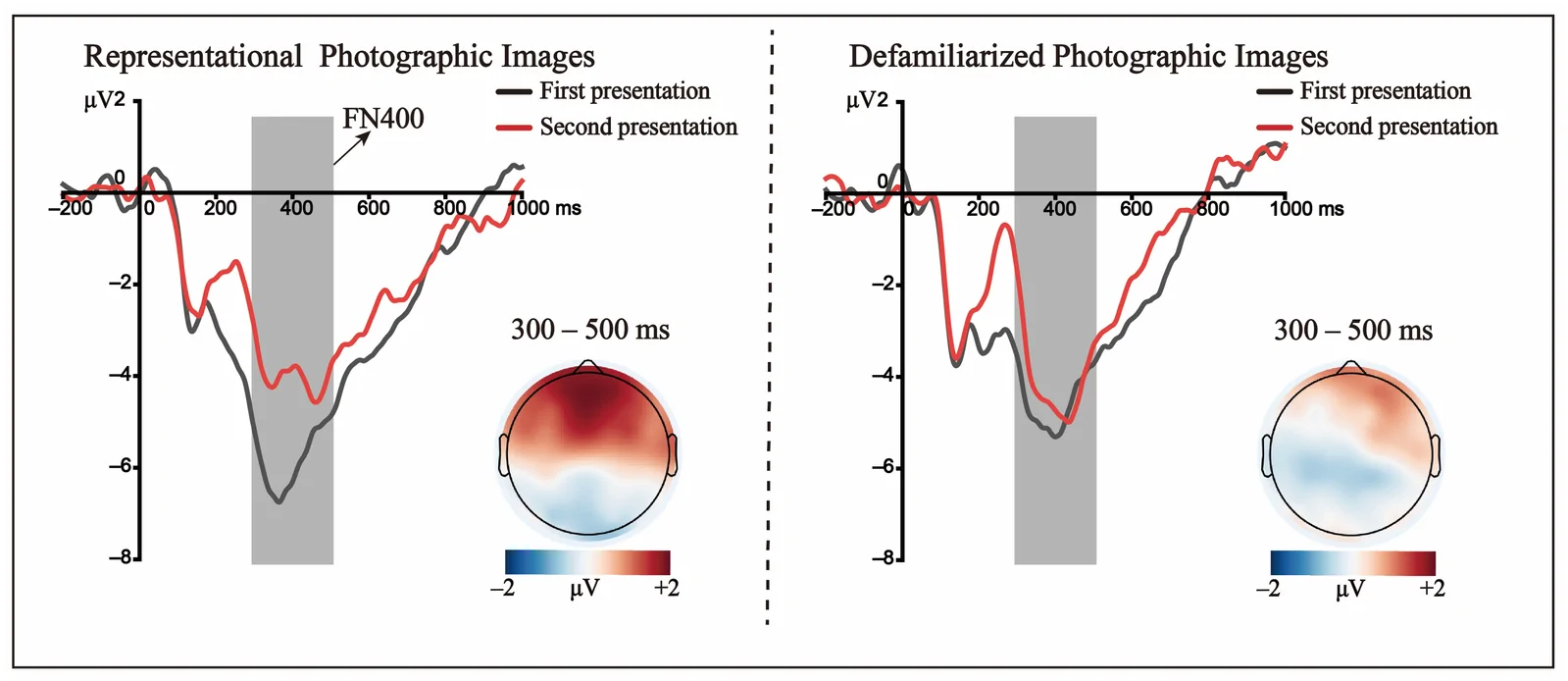
Grand-average ERP waveforms and topographical maps elicited by representational and defamiliarized photographic images across all participants. FN400 analyses were conducted at frontal electrodes (AF3, AF4, F1, Fz, F2). The (**left**) and (**right**) panels show the FN400 waveforms and topographical maps elicited by representational and defamiliarized photographic images, respectively; the black and red lines represent the mean FN400 amplitudes for the first and repeated presentations, respectively. The color scale of the topographical maps depicts the scalp distribution of FN400 amplitudes, with warm colors indicating more positive amplitudes and cool colors indicating more negative amplitudes. The shaded area represents the 300–500 ms time window, corresponding to the FN400 component.

**Figure 4:**
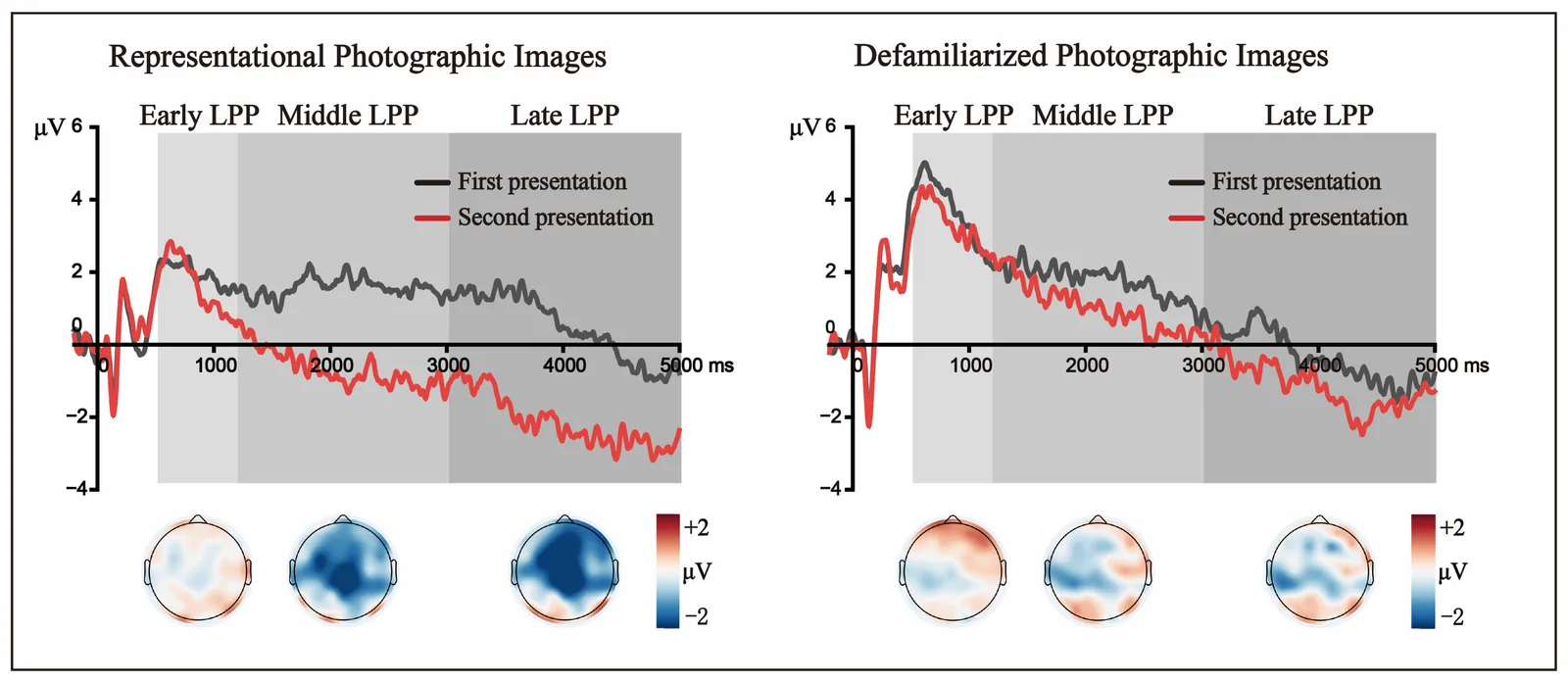
Late positive potential (LPP) components: grand-average ERP waveforms elicited by representational and defamiliarized photographic images across all participants. LPP mean amplitudes were recorded at electrodes C1, CZ, C2, CP1, CPZ, and CP2, and were analyzed across three consecutive time windows: 500–1200 ms (early LPP), 1200–3000 ms (middle LPP), and 3000–5000 ms (late LPP).

**Table 1:** Descriptive statistics for behavioral measures and ERP components.

	Presented Once		Presented Again	
	Representational Photographic Images	Defamiliarized Photographic Images	Representational Photographic Images	Defamiliarized Photographic Images
Proportion of trials judged as beautiful	0.59 ± 0.19	0.30 ± 0.31	0.57 ± 0.20	0.31 ± 0.31
FN400	−5.60 ± 6.32	−4.68 ± 6.36	−3.85 ± 6.13	−3.95 ± 6.04
Early LPP	2.01 ± 4.08	3.72 ± 4.12	1.84 ± 3.84	3.58 ± 4.38
Middle LPP	1.58 ± 3.95	1.47 ± 4.34	−0.57 ± 3.92	0.97 ± 4.25
Late LPP	0.46 ± 4.66	−0.77 ± 4.70	−2.05 ± 4.37	−1.37 ± 4.59

Note: Data are presented as mean ± SD. Behavioral data represent the proportion of trials judged as beautiful (range: 0–1). ERP component amplitudes are expressed in microvolts (µV).

**Table 2:** Results of the Trial-Level Binomial Generalized Linear Mixed-Effects Model for “Beautiful” Judgments.

Fixed Effects	β	SE	z/t	*p*	OR	95% CI
intercept	−0.514	0.211	−2.44	0.015	0.60	[0.40, 0.90]
condition	1.338	0.256	5.22	<0.001	3.81	[2.31, 6.30]
presentation order	0.119	0.256	0.46	0.643	1.13	[0.68, 1.86]
Condition × Presentation Order	−0.230	0.362	−0.64	0.525	0.79	[0.39, 1.61]

Note. The model used a binomial distribution with a logit link function; fixed effects included stimulus type, presentation order, and their interaction, with participants and image items included as random intercepts. The reference condition was “defamiliarized photographic images × initial presentation.”.

## Data Availability

The data presented in this study are available on request from the corresponding author due to privacy and institutional restrictions.

## Abbreviations

The following abbreviations are used in this manuscript:

ERPs	Event-Related Potentials
LPP	Late positive potential
DPI	Defamiliarized photographic images
RPI	Representational photographic images
